# Vitamin K supplementation and cardiovascular risk factors: a critical appraisal

**DOI:** 10.1017/jns.2024.22

**Published:** 2024-08-02

**Authors:** Aimen Nadeem, Zain Ali Nadeem, Umar Akram

**Affiliations:** 1 Department of Medicine, King Edward Medical University, Lahore, Punjab, Pakistan; 2 Department of Medicine, Allama Iqbal Medical College, Lahore, Punjab, Pakistan

**Keywords:** Bias, Cardiovascular risk factors, Certainty of evidence, Vitamin K

We read with pleasure the systematic review and meta-analysis by Zhao *et al.* ^(1)^ which provided valuable insight into the role of vitamin K supplementation in risk factors associated with cardiovascular disease. The authors did a great effort in their meta-analysis to clearly exhibit any effect, or lack thereof, of vitamin K supplementation on blood glucose levels, HbA1c, insulin resistance, homeostatic model assessment insulin resistance (HOMA-IR), body weight, body mass index (BMI), low-density lipoprotein (LDL) and high-density lipoprotein (HDL) cholesterol, C-reactive protein (CRP), and blood pressure. The findings are certainly interesting, but we find it necessary to point out a few inconsistencies in the methods and results that could potentially mislead or confuse the readers.

In the results, the authors have mentioned that ‘HOMA-IR was significantly reduced following vitamin K supplementation compared (WMD: –0.24, 95% confidence interval (CI): –0.49, –0.02, *P* = 0.047) with placebo”, but the forest plot displays the upper bound of the 95% CI as 0.02. This crosses the line of no effect, which is 0 in the case of continuous outcomes.^(2)^ We tried to resolve this ambiguity by pooling the data provided by the authors and obtained the same result. The authors seem to have misinterpreted the forest plot for HOMA-IR.

Additionally, the authors used the NutriGrade scoring system to evaluate the certainty of evidence.^(3)^ However, we found some oversight in its application. First, this scoring system is based on seven items, one of which is publication bias, as mentioned by the authors in their methods section. However, they have not described the methods employed to assess the publication bias and no such assessment is presented in the results. We sought to identify publication bias by constructing funnel plots for outcomes with more than ten studies^(4)^ and Doi plots with the associated Luis Furuya-Kanamori (LFK) index for articles with less than ten studies^(5)^ using the data provided. While we found no publication bias in the outcomes of glucose, total and LDL cholesterol, and systolic blood pressure, we observed sufficient evidence for publication bias in the remaining outcomes: insulin (LFK index = –1.72, minor asymmetry), HbA1c (LFK index = –4.06, major asymmetry), HOMA-IR (LFK index = –2.87, major asymmetry), weight (LFK index = 8.14 major asymmetry), BMI (LFK index = 4.5, major asymmetry), HDL cholesterol (LFK index = –1.5, minor asymmetry), triglycerides (LFK index = –1.02, minor asymmetry), CRP (LFK index = –1.44, minor asymmetry), and diastolic blood pressure (LFK index = –1.31, minor asymmetry). Second, the NutriGrade system is a tool to judge the certainty of evidence with regard to individual outcomes, classifying a particular outcome as high-, moderate-, low-, or very-low-quality evidence.^(3)^ The authors seem to have misunderstood the scoring system; while assessing the quality of a meta-analysis is useful in certain circumstances, NutriGrade is unsuitable for the task.

We commend Zhao *et al.* ^(1)^ for their valuable contribution and invite them to clarify the misinterpreted outcome. Moreover, we request that the authors reassess the certainty of evidence, keeping the potential publication bias in view and evaluate each outcome separately to help the readers better comprehend their results. Lastly, we urge the readers to exercise diligence when interpreting the findings.
